# Crystal structure and void analysis of tris­(2-amino-1-methyl­benzimidazolium) hexa­kis­(nitrato-κ^2^ *O*,*O*′)lanthanate(III)

**DOI:** 10.1107/S2056989022005163

**Published:** 2022-05-20

**Authors:** Bakhtigul Ruzieva, Rishad Kunafiev, Zukhra Kadirova, Shahlo Daminova

**Affiliations:** a Uzbekistan-Japan Innovation Center of Youth, University street 2B, Tashkent 100095, Uzbekistan; b National University of Uzbekistan named after Mirzo Ulugbek, University street 4, Tashkent 100174, Uzbekistan; c State Key Laboratory of Applied Organic Chemistry (SKLAOC), College of Chemistry and Chemical Engineering, Lanzhou University, Lanzhou 730000, People’s Republic of China; Vienna University of Technology, Austria

**Keywords:** benzimidazolium, lanthanum complex, crystal structure

## Abstract

The organic–inorganic complex salt, (C_8_H_10_N_3_)_3_[La(NO_3_)_6_], comprises a network of N-protonated 2-amino-1-methyl­benzimidazolium cations and hexa­kis­(nitrato)lanthanate(III) anions. The La^III^ atom is twelve-coordinate within a distorted icosa­hedral environment. In the unit cell, each pair of the La^III^ atoms lie nearly on one of the crystallographic glide planes. In the crystal structure, there are several N—H⋯O hydrogen-bonding inter­actions between the cations and terminal oxygen atoms from the nitrate moieties of the [La(NO_3_)_6_]^3–^ anion. Additional weak C—H⋯O hydrogen bonds between the cations and anions consolidate the three-dimensional arrangement of the structure. A packing analysis was performed to check the strength of the crystal packing.

## Abstract 2

The title hybrid lanthanum complex comprises an icosa­hedrally arranged La(NO_3_)_6_]^3–^ anion that is linked to the organic C_8_H_10_N_3_^+^ cations through N—H⋯O and C—H⋯O inter­actions.

## 1. Chemical context

Layered lanthanide complexes in the solid state or in solution often represent an one-dimensional transition-metal self-assembly (Chen *et al.*, 2017 ▸), frequently incorporated within functional groups from various ligand systems. These complexes not only provide excellent opportunities to widen the research scope of rare-earth compounds, but also feature a novel nuclear secondary building unit (SBU), forming porous and intrinsically electrically conductive structures (Skorupskii & Dincă, 2020 ▸). Although lanthanide ions have characteristic electronic configurations with their complexes being ideal candidates for new crystal structures and potential applications in superconductivity, magnetism, optics, electronics and catalysis (Eliseeva & Bünzli, 2010 ▸; Woodruff *et al.*, 2013 ▸), lanthanide complexes, especially polynuclear clusters, are not well understood (Barry *et al.*, 2016 ▸). Some reasons for this are the uncontrollable polynuclear arrangement of lanthanide complexes and the nature of lanthanide ions, with their high coordination numbers, kinetic instabilities, uncertain preferred stereochemistry, and the variable nature of their coordination spheres.

In this context, originally trying to isolate polynuclear mixed-ligand lanthanum complexes, we have isolated the title organic–inorganic complex lanthanum salt, 3C_8_H_10_N_3_^+^·[La(NO_3_)_6_]^3-^, tris­(2-amino-1-methyl­benz­im­id­az­olium)hexa­kis­(nitrato-O,O′)-lanthanate(III), (**1**), and report here its crystal structure and void analysis. 
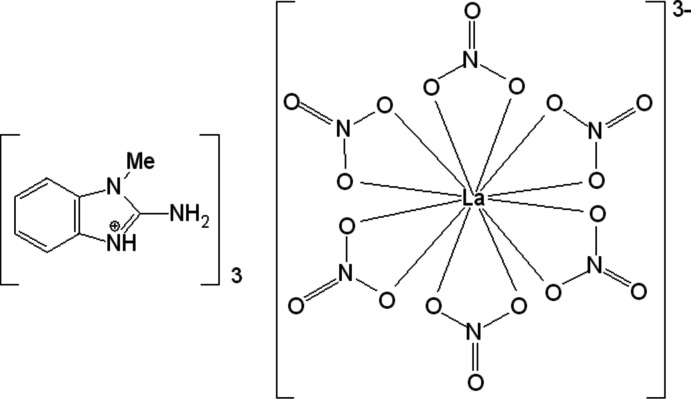


## 2. Structural commentary

**Figure 1:**
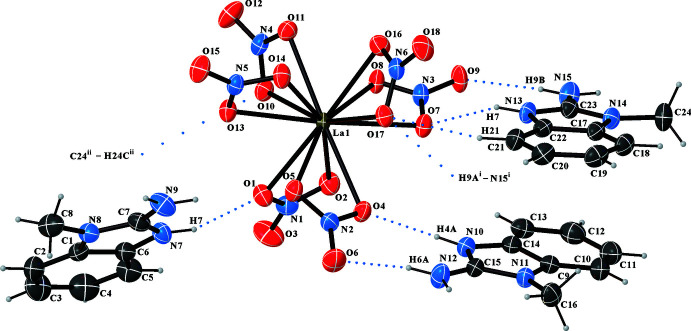
The mol­ecular structure of the [La(NO_3_)_6_]^3–^ anion and surrounding C_8_H_10_N_3_^+^ cations in (**1**), showing the atom-labeling scheme. Atomic displacement parameters are drawn at the 30% probability level and H atoms are shown at small spheres of arbitrary radius. Hydrogen bonds are shown as blue dotted lines. [Symmetry codes: (i) *x*, −*y* + 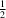 , *z* – 1/2; (ii) *x* − 1, −*y* + 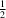 , *z* – 1/2.]

The La^III^ atom in (**1**) (Fig. 1 ▸) is twelve-coordinate by O atoms of the nitrato ligands with La—O bond lengths varying between 2.612 (2) and 2.707 (2) Å (Table 1 ▸). The nitrato ligands in the resulting [La(NO_3_)_6_]^3–^ anion surround the La^III^ atom in a highly distorted icosa­hedral environment. Bond lengths and angles in the [La(NO_3_)_6_]^3–^ anion show no significant deviations from those of other structures where the La^III^ atom is coordinated by nitrate anions and/or water mol­ecules (Drew *et al.*, 1998 ▸; Fowkes & Harrison, 2006 ▸; Skelton *et al.*, 2019 ▸; Polyzou *et al.*, 2012 ▸; Bezzubov *et al.*, 2017 ▸).

In the unit-cell of (**1**), each pair of La^III^ atoms nearly lie on each of the crystallographic glide planes [with deviations from the mean planes of 0.00 (7)–0.02 (1) Å]. The inter­section between the La^III^ atoms lying on neighboring glide planes at distances of 12.676 and 14.212 Å, respectively, passes through the center of inversion of the unit-cell.

## 3. Supra­molecular features

**Figure 2:**
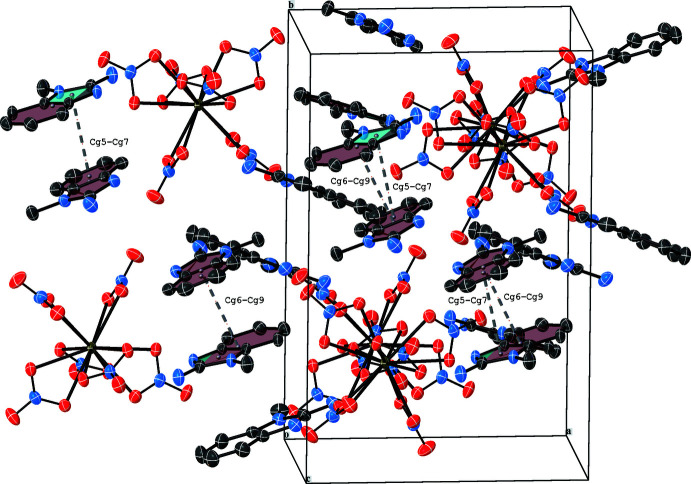
π—π stacking in the crystal structure of (**1**).

**Figure 3:**
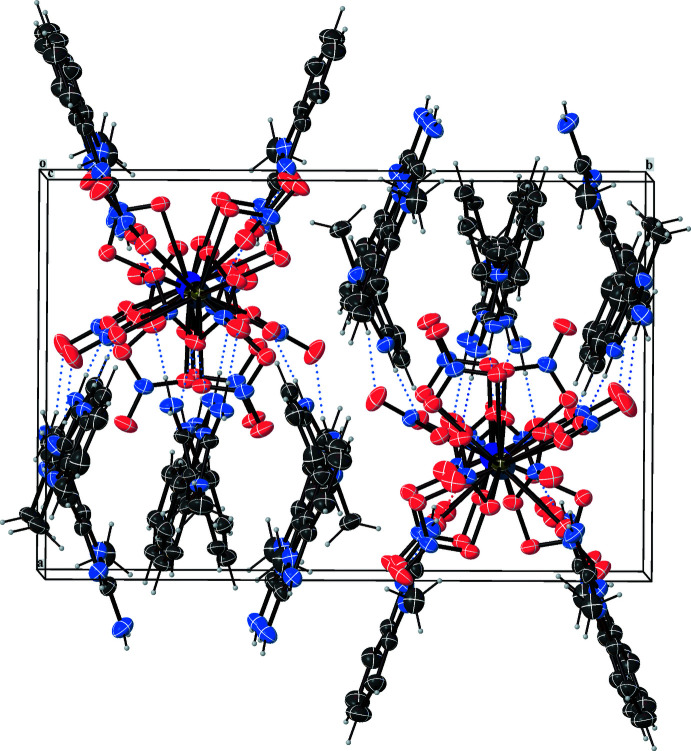
View of the crystal structure of (**1**) along [001], showing N—H⋯O and C—H⋯O hydrogen bonds drawn as blue dotted lines.

**Figure 4:**
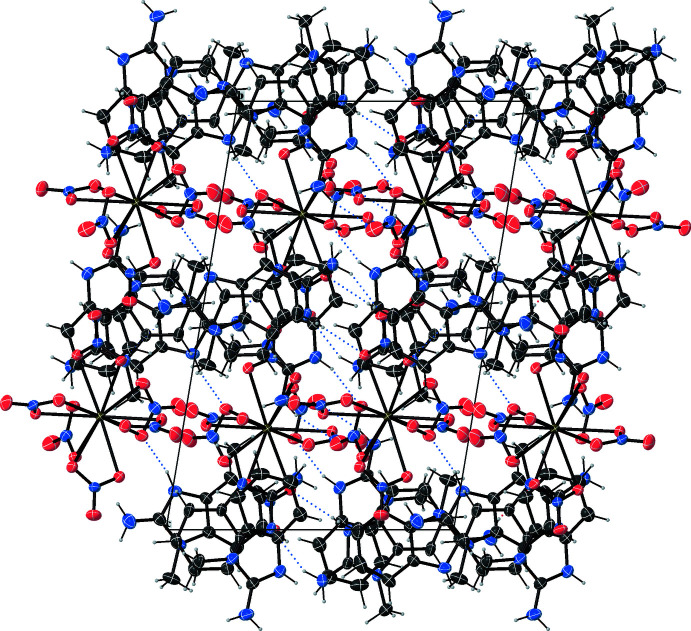
View of the crystal structure of (**1**) along [010], showing N—H⋯O and C—H⋯O hydrogen bonds drawn as blue dotted lines.

In the crystal structure of (**1**) the nitrate groups coordinate bidentately to the La^III^ atom. The corresponding La—O—N—O planes are close to coplanar, *i.e.* deviate slightly from 180°. As illustrated in Fig. 2 ▸, adjacent benzimidazolium mol­ecules stabilize the [La(NO_3_)_6_]^3–^ anion by N—H⋯O inter­actions (Fig. 1 ▸, Table 2 ▸). This arrangement is consolidated by slipped π⋯π inter­actions between neighbouring benzimidazolium cations [*Cg*5⋯*Cg*7 = 3.4515 (1) and *Cg*6⋯*Cg*9 = 3.5038 (1) Å with slippages of 0.649 and 0.219; *Cg5* and *Cg7* are the centroids of the C9–C14 and N13/C22/C17/N14/C23 rings, *Cg6* and *Cg9* are the centroids of the N10/C14–C9/N11/C15 and N13/C22–C17/N14/C23 rings; Fig. 2 ▸]. In the structure of (**1**), apart from the N—H⋯O inter­actions, there are two weak C—H⋯O inter­actions (Table 2 ▸) between adjacent [La(NO_3_)_6_]^3–^ anions and C_8_H_10_N_3_^+^ cations (Fig. 3 ▸). The three-dimensional network of (**1**) is assembled from all these inter­molecular contacts and inter­actions (Fig. 4 ▸).

## 4. Void analysis

**Figure 5:**
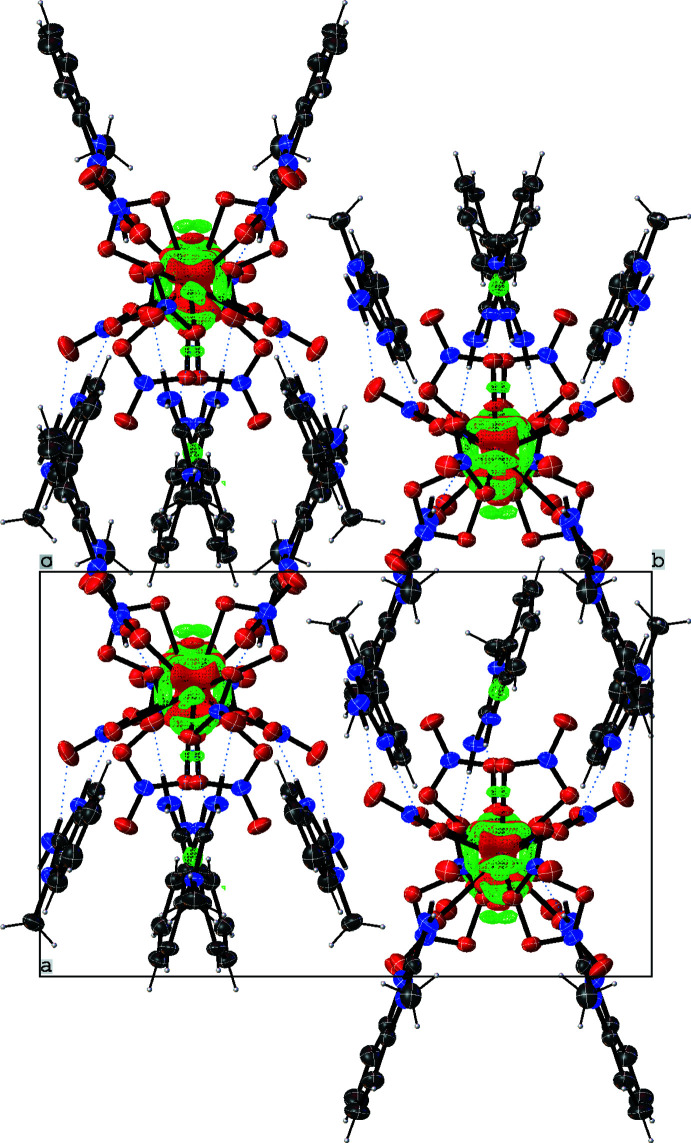
The electron density map of (**1**) in a view along [001].

**Figure 6:**
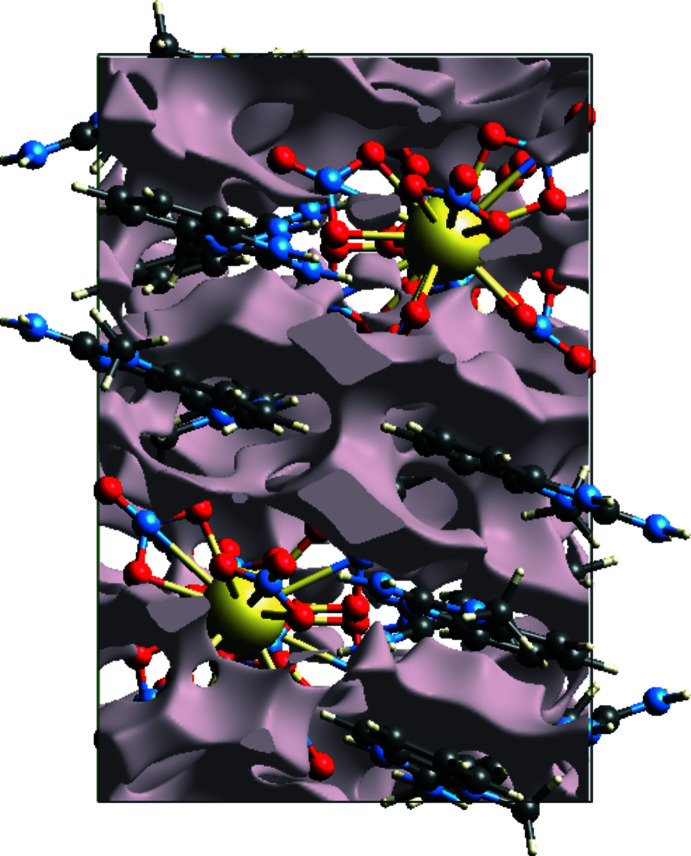
The void surface packing of (**1**) in a view along [001].

Mol­ecular surfaces can be used to quite accurately define the size and shape of a mol­ecule, and to visualize the space belonging to a mol­ecule in a crystal. To check whether the title compound is densely packed or not, a void-space analysis was performed. Based on isosurfaces of the procrystal electron density and electron-density mapping (Fig. 5 ▸), we have used the conventional approach of mapping void space by rolling a probe sphere of variable radius over a fused-sphere representation to locate and visualize the void space in a crystalline material, as well as readily compute surface areas and void volumes (Spackman *et al.*, 2021 ▸; Turner *et al.*, 2011 ▸). Fig. 6 ▸ shows the unit-cell packing for the title complex with a 0.002 a.u. void surface, and a volume of 388.80 Å^3^ per unit cell. This result indicates that voids occupy 10.7% of the space and, hence, the mol­ecules can be considered as densely packed in the crystal of (**1**).

## 5. Database survey

The structure of the mol­ecular [La(NO_3_)_6_]^3–^ anion was first reported by Drew *et al.* (1998 ▸). A search of the Cambridge Structural Database (CSD, version 5.42, update of September 2021; Groom *et al.*, 2016 ▸) revealed that there are six other reports of this moiety. One was obtained from the synthesis of a dinuclear Ni^II^/La^III^ complex containing the rare-earth metal in separate ions (Polyzou *et al.*, 2012 ▸), the second in research into materials with luminescent properties for developing new drugs (Esteban-Parra *et al.*, 2020 ▸), the third is a lanthanum/peptide heterometallic complex with inter­esting optical properties (Bezzubov *et al.*, 2017 ▸), the forth was studied during synthesis and theoretical calculations at the DFT level of di-La complexes with a pendant-armed macrocycle (Fernández-Fernández *et al.*, 2006 ▸), the fifth is a heteronuclear nitrato lanthanide complex with inter­esting magnetic properties (Thatipamula *et al.*, 2019 ▸), and the sixth is a pyridine imidazolium lanthanum complex (Skelton *et al.*, 2019 ▸). The crystal structure of the last compound comprises the anionic unit as ideal [La(NO_3_)_6_]^3–^, *i.e.* oppositely faced nitrate moieties lie co-planar to the La^III^ atom, forming a paddle-wheel-shaped structure. The latter is one of the most closely related structures to (**1**), with the main difference being the number of cations.

## 6. Synthesis and crystallization

10 ml of an ethanol solution of La(NO_3_)_3_·6H_2_O (216.8 mg, 0.0005 mmol) was stirred at room temperature for 1 h. Then a 10 ml ethanol solution of 2-amino-1-methyl­benzimidazole (220.5 mg, 0.0015 mmol) was gradually added dropwise to the stirring mixture over 50 min at 303 K. Immediately after this, the mixture was heated in a reflux condenser at boiling temperature for 30 min. The solution was filtered and allowed to cool. The obtained yellowish single crystalline product was washed several times in pure acetone and allowed to air-dry at room temperature.

## 7. Refinement

Crystal data, data collection and structure refinement details are summarized in Table 3 ▸. All hydrogen atoms were positioned geometrically with C—H = 0.93–0.96 Å and refined using a riding model with Uiso(H) = 1.5*U*_eq_(C) for methyl groups and 1.2*U*_eq_(C) for the other groups. Aromatic/amide hydrogen atoms were refined in a similar manner.

## Supplementary Material

Crystal structure: contains datablock(s) I. DOI: 10.1107/S2056989022005163/wm5638sup1.cif

Structure factors: contains datablock(s) I. DOI: 10.1107/S2056989022005163/wm5638Isup2.hkl

Supporting information file. DOI: 10.1107/S2056989022005163/wm5638sup3.txt

CCDC reference: 2132821

Additional supporting information:  crystallographic information; 3D view; checkCIF report

## Figures and Tables

**Table 1:** Selected geometric parameters (Å, °)

La1—O1	2.646 (2)	La1—O14	2.6551 (19)
La1—O2	2.707 (2)	La1—O16	2.674 (2)
La1—O4	2.650 (2)	La1—O17	2.662 (2)
La1—O5	2.661 (2)	N7—C6	1.387 (4)
La1—O7	2.699 (2)	N7—C7	1.335 (3)
La1—O8	2.6469 (18)	N8—C1	1.395 (3)
La1—O10	2.6520 (18)	N8—C7	1.341 (4)
La1—O11	2.631 (2)	N8—C8	1.455 (3)
La1—O13	2.612 (2)	N9—C7	1.313 (4)
			
O1—La1—O2	47.45 (6)	O11—La1—O10	47.98 (6)
O4—La1—O5	47.94 (6)	O13—La1—O14	48.26 (6)
O8—La1—O7	47.69 (6)	O17—La1—O16	47.80 (6)

**Table 2:** Hydrogen-bond geometry (Å, °)

*D*—H⋯*A*	*D*—H	H⋯*A*	*D*⋯*A*	*D*—H⋯*A*
C21—H21⋯O17	0.93	2.64	3.530 (5)	161
C24^i^—H24*C* ^i^⋯O10	0.96	2.52	3.348 (5)	138
N7—H7⋯O1	0.86	2.01	2.819 (3)	157
N10—H4*A*⋯O4	0.86	2.05	2.889 (3)	164
N12—H6*A*⋯O6	0.86	2.10	2.944 (3)	165
N13—H7⋯O7	0.86	2.11	2.920 (4)	156
N15—H9*B*⋯O9	0.86	2.14	2.946 (3)	155
N15^ii^—H9*A* ^ii^⋯O17	0.86	2.32	3.001 (3)	136

Symmetry codes: (i) 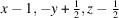 ; (ii) 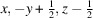 .

**Table 3:** Experimental details

Crystal data	
Chemical formula	(C_8_H_10_N_3_)_3_[La(NO_3_)_6_]
*M*_r_	955.54
Crystal system, space group	Monoclinic, *P*2_1_/*c*
Temperature (K)	293
*a*, *b*, *c* (Å)	11.78754 (10), 17.59536 (14), 17.79338 (15)
β (°)	99.4928 (8)
*V* (Å^3^)	3639.92 (5)
*Z*	4
Radiation type	Cu *K*α
μ (mm^−1^)	9.95
Crystal size (mm)	0.21 × 0.18 × 0.12

Data collection	
Diffractometer	XtaLAB Synergy, Single source at home/near, HyPix3000
Absorption correction	Multi-scan (*CrysAlis PRO*; Rigaku OD, 2020 ▸)
*T*_min_, *T*_max_	0.281, 1.000
No. of measured, independent and observed [*I* > 2σ(*I*)] reflections	21842, 7018, 6305
*R*_int_	0.038
(sin θ/λ)_max_ (Å^−1^)	0.615

Refinement	
*R*[*F* ^2^ > 2σ(*F* ^2^)], *wR*(*F* ^2^), *S*	0.032, 0.089, 1.05
No. of reflections	7018
No. of parameters	527
H-atom treatment	H-atom parameters constrained
Δρ_max_, Δρ_min_ (e Å^−3^)	0.69, −0.73

Computer programs: *CrysAlis PRO* (Rigaku OD, 2020 ▸), *SHELXS* (Sheldrick, 2008 ▸), *SHELXL* (Sheldrick, 2015 ▸), *ORTEP-3 for Windows* (Farrugia, 2012 ▸), *Mercury* (Macrae *et al.*, 2020 ▸), *OLEX2* (Dolomanov *et al.*, 2009 ▸), *PLATON* (Spek, 2020 ▸) and *publCIF* (Westrip, 2010 ▸).

## supplementary crystallographic information

### Crystal data

**Table:**

(C_8_H_10_N_3_)_3_[La(NO_3_)_6_]	*F*(000) = 1920
*M**_r_* = 955.54	*D*_x_ = 1.744 Mg m^−^^3^
Monoclinic, *P*2_1_/*c*	Cu *K*α radiation, λ = 1.54184 Å
*a* = 11.78754 (10) Å	Cell parameters from 13771 reflections
*b* = 17.59536 (14) Å	θ = 2.5–71.2°
*c* = 17.79338 (15) Å	µ = 9.95 mm^−^^1^
β = 99.4928 (8)°	*T* = 293 K
*V* = 3639.92 (5) Å^3^	Block, yellow
*Z* = 4	0.21 × 0.18 × 0.12 mm

### Data collection

**Table:**

XtaLAB Synergy, Single source at home/near, HyPix3000 diffractometer	7018 independent reflections
Radiation source: micro-focus sealed X-ray tube, PhotonJet (Cu) X-ray Source	6305 reflections with *I* > 2σ(*I*)
Mirror monochromator	*R*_int_ = 0.038
Detector resolution: 10.0000 pixels mm^-1^	θ_max_ = 71.4°, θ_min_ = 3.6°
ω scans	*h* = −14→10
Absorption correction: multi-scan (*CrysAlisPro*; Rigaku OD, 2020)	*k* = −21→21
*T*_min_ = 0.281, *T*_max_ = 1.000	*l* = −21→21
21842 measured reflections	

### Refinement

**Table:**

Refinement on *F*^2^	Hydrogen site location: inferred from neighbouring sites
Least-squares matrix: full	H-atom parameters constrained
*R*[*F*^2^ > 2σ(*F*^2^)] = 0.032	*w* = 1/[σ^2^(*F*_o_^2^) + (0.054*P*)^2^] where *P* = (*F*_o_^2^ + 2*F*_c_^2^)/3
*wR*(*F*^2^) = 0.089	(Δ/σ)_max_ = 0.002
*S* = 1.05	Δρ_max_ = 0.69 e Å^−^^3^
7018 reflections	Δρ_min_ = −0.73 e Å^−^^3^
527 parameters	Extinction correction: SHELXL (Sheldrick, 2015a), Fc^*^=kFc[1+0.001xFc^2^λ^3^/sin(2θ)]^-1/4^
0 restraints	Extinction coefficient: 0.00063 (4)

### Special details

**Table:**

Geometry. All esds (except the esd in the dihedral angle between two l.s. planes) are estimated using the full covariance matrix. The cell esds are taken into account individually in the estimation of esds in distances, angles and torsion angles; correlations between esds in cell parameters are only used when they are defined by crystal symmetry. An approximate (isotropic) treatment of cell esds is used for estimating esds involving l.s. planes.

### Fractional atomic coordinates and isotropic or equivalent isotropic displacement parameters (Å^2^)

**Table:**

	*x*	*y*	*z*	*U*_iso_*/*U*_eq_	
La1	0.29343 (2)	0.24596 (2)	0.26399 (2)	0.03257 (8)	
O1	0.13909 (18)	0.35278 (13)	0.21917 (11)	0.0522 (5)	
O2	0.16522 (19)	0.33582 (13)	0.34071 (11)	0.0586 (5)	
O3	0.0273 (2)	0.40812 (16)	0.28696 (17)	0.0880 (9)	
O4	0.39500 (18)	0.38018 (12)	0.28782 (11)	0.0498 (5)	
O5	0.35777 (17)	0.34828 (12)	0.16921 (10)	0.0482 (5)	
O6	0.4493 (2)	0.45295 (14)	0.20134 (14)	0.0794 (7)	
O7	0.4095 (2)	0.25349 (10)	0.40779 (14)	0.0475 (6)	
O8	0.25431 (15)	0.18594 (12)	0.39370 (10)	0.0487 (5)	
O9	0.37164 (18)	0.18384 (14)	0.50096 (11)	0.0627 (6)	
O10	0.07926 (15)	0.19647 (12)	0.25603 (11)	0.0484 (5)	
O11	0.20210 (16)	0.10968 (12)	0.24177 (11)	0.0483 (5)	
O12	0.0197 (2)	0.08323 (15)	0.22181 (14)	0.0737 (7)	
O13	0.19323 (18)	0.22778 (13)	0.12292 (12)	0.0480 (5)	
O14	0.36267 (16)	0.17855 (13)	0.14595 (11)	0.0530 (5)	
O15	0.2596 (2)	0.16638 (15)	0.03340 (11)	0.0679 (6)	
O16	0.44669 (18)	0.13478 (12)	0.30224 (12)	0.0528 (5)	
O17	0.52066 (19)	0.24035 (11)	0.27129 (13)	0.0468 (5)	
O18	0.62845 (18)	0.14095 (15)	0.29585 (13)	0.0683 (6)	
N1	0.1087 (2)	0.36713 (14)	0.28342 (15)	0.0511 (6)	
N2	0.40091 (19)	0.39556 (13)	0.21858 (13)	0.0460 (5)	
N3	0.34526 (18)	0.20720 (15)	0.43578 (12)	0.0435 (5)	
N4	0.0971 (2)	0.12845 (15)	0.23923 (13)	0.0453 (6)	
N5	0.27197 (19)	0.18996 (14)	0.09850 (12)	0.0426 (5)	
N6	0.53406 (19)	0.17059 (14)	0.29008 (12)	0.0449 (5)	
N7	−0.0092 (2)	0.40624 (14)	0.08932 (13)	0.0515 (6)	
H1	0.029959	0.401296	0.134312	0.062*	
N8	−0.0554 (2)	0.40492 (13)	−0.03455 (13)	0.0477 (5)	
N9	0.1321 (2)	0.36555 (16)	0.01938 (17)	0.0666 (8)	
H3A	0.181714	0.358860	0.060033	0.080*	
H3B	0.150431	0.356204	−0.024523	0.080*	
C1	−0.1515 (2)	0.43169 (16)	−0.00624 (16)	0.0457 (6)	
C2	−0.2576 (3)	0.4554 (2)	−0.0427 (2)	0.0644 (9)	
H2	−0.277566	0.454118	−0.095461	0.077*	
C3	−0.3333 (3)	0.4813 (3)	0.0036 (3)	0.0831 (13)	
H3	−0.405826	0.498305	−0.018592	0.100*	
C4	−0.3035 (3)	0.4826 (3)	0.0817 (3)	0.0828 (12)	
H4	−0.356791	0.500237	0.110746	0.099*	
C5	−0.1968 (3)	0.4584 (2)	0.1188 (2)	0.0645 (9)	
H5	−0.177024	0.459587	0.171542	0.077*	
C6	−0.1216 (2)	0.43237 (16)	0.07252 (16)	0.0458 (6)	
C7	0.0283 (3)	0.39002 (16)	0.02434 (16)	0.0485 (7)	
C8	−0.0481 (3)	0.3937 (2)	−0.11461 (17)	0.0681 (10)	
H8A	−0.123423	0.383637	−0.142553	0.102*	
H8B	0.001541	0.351474	−0.119738	0.102*	
H8C	−0.017603	0.438752	−0.134365	0.102*	
N10	0.5796 (2)	0.43538 (14)	0.40202 (13)	0.0487 (6)	
H4A	0.516816	0.419368	0.375155	0.058*	
N11	0.7521 (2)	0.48326 (14)	0.43189 (14)	0.0476 (6)	
N12	0.6710 (2)	0.47912 (16)	0.30120 (14)	0.0631 (7)	
H6A	0.613623	0.466271	0.267181	0.076*	
H6B	0.730645	0.499816	0.287798	0.076*	
C9	0.7153 (3)	0.46337 (17)	0.50060 (18)	0.0475 (7)	
C10	0.7675 (3)	0.4709 (2)	0.57513 (19)	0.0610 (8)	
H10	0.840999	0.491168	0.588073	0.073*	
C11	0.7050 (4)	0.4467 (2)	0.62983 (19)	0.0703 (10)	
H11	0.736881	0.451331	0.681041	0.084*	
C12	0.5960 (3)	0.4157 (2)	0.6103 (2)	0.0671 (9)	
H12	0.556988	0.399753	0.648861	0.081*	
C13	0.5436 (3)	0.40784 (18)	0.53563 (19)	0.0566 (7)	
H13	0.470361	0.387121	0.522711	0.068*	
C14	0.6059 (2)	0.43245 (16)	0.48098 (16)	0.0468 (6)	
C15	0.6674 (2)	0.46703 (16)	0.37422 (16)	0.0468 (6)	
C16	0.8664 (3)	0.5101 (2)	0.4250 (2)	0.0626 (8)	
H16A	0.922846	0.475477	0.450741	0.094*	
H16B	0.878371	0.559568	0.447659	0.094*	
H16C	0.873705	0.512912	0.372153	0.094*	
N13	0.6355 (2)	0.23763 (14)	0.50237 (16)	0.0445 (6)	
H7	0.572120	0.228425	0.472247	0.053*	
N14	0.7613 (2)	0.24800 (12)	0.60674 (17)	0.0454 (6)	
N15	0.5738 (2)	0.21128 (17)	0.61913 (15)	0.0601 (7)	
H9A	0.591261	0.208013	0.667896	0.072*	
H9B	0.504897	0.201377	0.596973	0.072*	
C17	0.8178 (3)	0.26746 (17)	0.54597 (19)	0.0428 (6)	
C18	0.9309 (2)	0.28901 (19)	0.54492 (19)	0.0532 (7)	
H18	0.985120	0.291954	0.589167	0.064*	
C19	0.9581 (3)	0.30592 (19)	0.4736 (2)	0.0596 (8)	
H19	1.032729	0.320673	0.469900	0.071*	
C20	0.8769 (3)	0.30138 (19)	0.40783 (19)	0.0573 (8)	
H20	0.898241	0.313790	0.361311	0.069*	
C21	0.7647 (3)	0.2788 (2)	0.40965 (18)	0.0520 (7)	
H21	0.710398	0.275564	0.365475	0.062*	
C22	0.7376 (3)	0.26148 (15)	0.4800 (2)	0.0423 (7)	
C23	0.6521 (3)	0.23136 (18)	0.57861 (18)	0.0447 (6)	
C24	0.8111 (4)	0.2469 (2)	0.6871 (2)	0.0665 (11)	
H24A	0.849109	0.199223	0.699414	0.100*	
H24B	0.751263	0.253337	0.717210	0.100*	
H24C	0.865729	0.287569	0.697737	0.100*	

### Atomic displacement parameters (Å^2^)

**Table:**

	*U*^11^	*U*^22^	*U*^33^	*U*^12^	*U*^13^	*U*^23^
La1	0.02761 (11)	0.04264 (11)	0.02672 (11)	−0.00091 (5)	0.00235 (7)	−0.00063 (5)
O1	0.0533 (12)	0.0610 (13)	0.0427 (11)	0.0138 (10)	0.0095 (9)	0.0013 (9)
O2	0.0657 (13)	0.0691 (14)	0.0419 (11)	0.0064 (11)	0.0119 (10)	−0.0032 (10)
O3	0.0832 (18)	0.0851 (19)	0.103 (2)	0.0438 (15)	0.0375 (16)	0.0091 (16)
O4	0.0563 (12)	0.0531 (12)	0.0378 (10)	−0.0101 (10)	0.0015 (9)	−0.0034 (9)
O5	0.0492 (11)	0.0588 (12)	0.0360 (10)	−0.0085 (10)	0.0051 (8)	−0.0045 (9)
O6	0.0942 (18)	0.0657 (15)	0.0731 (16)	−0.0359 (14)	−0.0013 (13)	0.0180 (12)
O7	0.0387 (12)	0.0676 (15)	0.0356 (12)	−0.0118 (8)	0.0045 (9)	0.0017 (8)
O8	0.0391 (10)	0.0704 (14)	0.0345 (9)	−0.0124 (9)	−0.0006 (8)	0.0021 (9)
O9	0.0600 (12)	0.0911 (17)	0.0328 (10)	−0.0130 (12)	−0.0048 (9)	0.0158 (10)
O10	0.0362 (10)	0.0572 (13)	0.0513 (11)	−0.0035 (9)	0.0059 (8)	−0.0069 (9)
O11	0.0439 (11)	0.0488 (12)	0.0517 (12)	−0.0018 (8)	0.0064 (9)	−0.0041 (8)
O12	0.0621 (14)	0.0842 (18)	0.0775 (16)	−0.0371 (13)	0.0193 (12)	−0.0255 (14)
O13	0.0387 (11)	0.0629 (12)	0.0410 (11)	0.0030 (10)	0.0027 (9)	−0.0057 (10)
O14	0.0379 (10)	0.0772 (15)	0.0426 (11)	0.0085 (10)	0.0027 (8)	−0.0074 (10)
O15	0.0750 (14)	0.0911 (18)	0.0375 (11)	−0.0059 (13)	0.0091 (10)	−0.0185 (11)
O16	0.0480 (12)	0.0536 (12)	0.0560 (12)	0.0014 (10)	0.0067 (9)	0.0066 (10)
O17	0.0366 (11)	0.0610 (13)	0.0425 (12)	0.0019 (8)	0.0053 (9)	0.0045 (8)
O18	0.0436 (11)	0.0835 (17)	0.0754 (15)	0.0232 (11)	0.0027 (10)	−0.0033 (13)
N1	0.0535 (15)	0.0479 (14)	0.0550 (15)	0.0062 (12)	0.0182 (12)	−0.0045 (11)
N2	0.0436 (12)	0.0445 (13)	0.0481 (13)	−0.0035 (10)	0.0023 (10)	0.0034 (10)
N3	0.0361 (11)	0.0619 (15)	0.0319 (11)	−0.0024 (11)	0.0041 (9)	0.0000 (10)
N4	0.0435 (13)	0.0570 (15)	0.0357 (12)	−0.0119 (11)	0.0073 (10)	−0.0055 (10)
N5	0.0425 (12)	0.0542 (14)	0.0312 (11)	−0.0076 (11)	0.0062 (9)	−0.0023 (9)
N6	0.0398 (12)	0.0600 (15)	0.0330 (11)	0.0067 (11)	0.0006 (9)	−0.0030 (10)
N7	0.0584 (14)	0.0567 (15)	0.0389 (12)	0.0078 (12)	0.0070 (11)	0.0049 (11)
N8	0.0585 (14)	0.0471 (13)	0.0398 (12)	0.0029 (11)	0.0149 (11)	−0.0002 (10)
N9	0.0623 (17)	0.0686 (18)	0.0737 (19)	0.0211 (14)	0.0248 (14)	0.0147 (15)
C1	0.0516 (16)	0.0414 (15)	0.0452 (15)	−0.0024 (12)	0.0106 (12)	−0.0032 (12)
C2	0.0538 (19)	0.075 (2)	0.062 (2)	−0.0026 (17)	0.0007 (16)	−0.0021 (18)
C3	0.045 (2)	0.097 (3)	0.108 (4)	0.010 (2)	0.013 (2)	0.002 (3)
C4	0.067 (2)	0.092 (3)	0.100 (3)	0.008 (2)	0.044 (2)	−0.003 (3)
C5	0.069 (2)	0.073 (2)	0.058 (2)	0.0051 (18)	0.0309 (17)	−0.0028 (17)
C6	0.0511 (16)	0.0450 (15)	0.0433 (15)	0.0008 (13)	0.0140 (12)	−0.0002 (12)
C7	0.0549 (17)	0.0433 (15)	0.0511 (16)	0.0071 (13)	0.0197 (14)	0.0069 (12)
C8	0.099 (3)	0.066 (2)	0.0451 (17)	−0.0033 (19)	0.0292 (18)	−0.0037 (15)
N10	0.0437 (12)	0.0484 (14)	0.0511 (14)	−0.0051 (11)	−0.0004 (10)	−0.0009 (11)
N11	0.0468 (13)	0.0479 (14)	0.0455 (13)	−0.0055 (11)	−0.0005 (10)	0.0030 (11)
N12	0.0683 (17)	0.0747 (19)	0.0436 (14)	−0.0184 (15)	0.0007 (12)	0.0021 (13)
C9	0.0527 (18)	0.0402 (15)	0.0479 (16)	0.0006 (13)	0.0035 (13)	0.0018 (12)
C10	0.064 (2)	0.063 (2)	0.0524 (18)	−0.0001 (17)	−0.0026 (15)	−0.0013 (15)
C11	0.097 (3)	0.066 (2)	0.0459 (18)	0.004 (2)	0.0034 (18)	0.0006 (16)
C12	0.083 (2)	0.061 (2)	0.062 (2)	0.0085 (19)	0.0247 (19)	0.0075 (16)
C13	0.0537 (17)	0.0481 (17)	0.070 (2)	0.0031 (14)	0.0154 (15)	0.0049 (15)
C14	0.0499 (15)	0.0374 (14)	0.0521 (16)	0.0049 (12)	0.0059 (13)	−0.0008 (12)
C15	0.0487 (15)	0.0430 (15)	0.0453 (15)	−0.0034 (12)	−0.0020 (12)	−0.0022 (12)
C16	0.0466 (16)	0.070 (2)	0.068 (2)	−0.0130 (16)	0.0000 (14)	0.0074 (17)
N13	0.0280 (12)	0.0551 (14)	0.0482 (15)	−0.0019 (10)	−0.0003 (11)	−0.0078 (11)
N14	0.0320 (13)	0.0571 (17)	0.0457 (15)	−0.0002 (9)	0.0028 (12)	−0.0058 (9)
N15	0.0407 (13)	0.087 (2)	0.0537 (15)	−0.0050 (14)	0.0110 (11)	−0.0069 (15)
C17	0.0326 (14)	0.0438 (14)	0.0507 (17)	0.0036 (12)	0.0032 (12)	−0.0056 (13)
C18	0.0317 (14)	0.060 (2)	0.0656 (19)	−0.0028 (13)	0.0024 (13)	−0.0112 (15)
C19	0.0440 (16)	0.061 (2)	0.076 (2)	−0.0082 (15)	0.0188 (16)	−0.0096 (17)
C20	0.0565 (18)	0.060 (2)	0.0582 (19)	−0.0075 (15)	0.0197 (15)	−0.0042 (15)
C21	0.0505 (17)	0.0574 (18)	0.0468 (17)	−0.0019 (15)	0.0038 (13)	−0.0063 (14)
C22	0.0318 (14)	0.0418 (15)	0.0535 (19)	0.0018 (11)	0.0073 (13)	−0.0078 (12)
C23	0.0335 (15)	0.0509 (15)	0.0484 (17)	0.0051 (13)	0.0032 (12)	−0.0047 (13)
C24	0.051 (2)	0.095 (3)	0.049 (2)	−0.0038 (16)	−0.0054 (17)	−0.0030 (15)

### Geometric parameters (Å, º)

**Table:**

La1—O1	2.646 (2)	C5—C6	1.383 (4)
La1—O2	2.707 (2)	C8—H8A	0.9600
La1—O4	2.650 (2)	C8—H8B	0.9600
La1—O5	2.661 (2)	C8—H8C	0.9600
La1—O7	2.699 (2)	N10—H4A	0.8600
La1—O8	2.6469 (18)	N10—C14	1.389 (4)
La1—O10	2.6520 (18)	N10—C15	1.339 (4)
La1—O11	2.631 (2)	N11—C9	1.407 (4)
La1—O13	2.612 (2)	N11—C15	1.339 (4)
La1—O14	2.6551 (19)	N11—C16	1.452 (4)
La1—O16	2.674 (2)	N12—H6A	0.8600
La1—O17	2.662 (2)	N12—H6B	0.8600
O1—N1	1.278 (3)	N12—C15	1.324 (4)
O2—N1	1.250 (3)	C9—C10	1.373 (4)
O3—N1	1.210 (3)	C9—C14	1.390 (4)
O4—N2	1.274 (3)	C10—H10	0.9300
O5—N2	1.255 (3)	C10—C11	1.381 (5)
O6—N2	1.223 (3)	C11—H11	0.9300
O7—N3	1.269 (3)	C11—C12	1.387 (5)
O8—N3	1.259 (3)	C12—H12	0.9300
O9—N3	1.221 (3)	C12—C13	1.377 (5)
O10—N4	1.260 (3)	C13—H13	0.9300
O11—N4	1.275 (3)	C13—C14	1.382 (4)
O12—N4	1.212 (3)	C16—H16A	0.9600
O13—N5	1.275 (3)	C16—H16B	0.9600
O14—N5	1.264 (3)	C16—H16C	0.9600
O15—N5	1.216 (3)	N13—H7	0.8600
O16—N6	1.256 (3)	N13—C22	1.393 (4)
O17—N6	1.275 (3)	N13—C23	1.343 (4)
O18—N6	1.218 (3)	N14—C17	1.403 (4)
N7—H1	0.8600	N14—C23	1.335 (4)
N7—C6	1.387 (4)	N14—C24	1.453 (5)
N7—C7	1.335 (3)	N15—H9A	0.8600
N8—C1	1.395 (3)	N15—H9B	0.8600
N8—C7	1.341 (4)	N15—C23	1.310 (4)
N8—C8	1.455 (3)	C17—C18	1.389 (4)
N9—H3A	0.8600	C17—C22	1.385 (5)
N9—H3B	0.8600	C18—H18	0.9300
N9—C7	1.313 (4)	C18—C19	1.392 (4)
C1—C2	1.375 (4)	C19—H19	0.9300
C1—C6	1.388 (4)	C19—C20	1.386 (5)
C2—H2	0.9300	C20—H20	0.9300
C2—C3	1.388 (5)	C20—C21	1.386 (4)
C3—H3	0.9300	C21—H21	0.9300
C3—C4	1.376 (6)	C21—C22	1.376 (5)
C4—H4	0.9300	C24—H24A	0.9600
C4—C5	1.386 (6)	C24—H24B	0.9600
C5—H5	0.9300	C24—H24C	0.9600
			
O1—La1—O2	47.45 (6)	H3A—N9—H3B	120.0
O1—La1—O4	71.65 (6)	C7—N9—H3A	120.0
O1—La1—O5	65.29 (6)	C7—N9—H3B	120.0
O1—La1—O7	117.83 (6)	C2—C1—N8	131.4 (3)
O1—La1—O8	109.65 (6)	C2—C1—C6	122.2 (3)
O1—La1—O10	67.42 (7)	C6—C1—N8	106.3 (3)
O1—La1—O14	111.39 (6)	C1—C2—H2	121.8
O1—La1—O16	177.08 (6)	C1—C2—C3	116.3 (4)
O1—La1—O17	132.64 (6)	C3—C2—H2	121.8
O4—La1—O2	70.97 (7)	C2—C3—H3	119.2
O4—La1—O5	47.94 (6)	C4—C3—C2	121.5 (4)
O4—La1—O7	70.09 (6)	C4—C3—H3	119.2
O4—La1—O10	134.59 (6)	C3—C4—H4	118.8
O4—La1—O14	109.57 (6)	C3—C4—C5	122.4 (3)
O4—La1—O16	110.05 (6)	C5—C4—H4	118.8
O4—La1—O17	66.47 (6)	C4—C5—H5	122.0
O5—La1—O2	99.53 (7)	C6—C5—C4	116.1 (3)
O5—La1—O7	114.50 (6)	C6—C5—H5	122.0
O5—La1—O16	113.90 (6)	N7—C6—C1	106.8 (2)
O5—La1—O17	70.51 (6)	C5—C6—N7	131.6 (3)
O7—La1—O2	74.41 (7)	C5—C6—C1	121.5 (3)
O8—La1—O2	66.50 (7)	N7—C7—N8	109.2 (2)
O8—La1—O4	110.80 (6)	N9—C7—N7	125.0 (3)
O8—La1—O5	158.64 (6)	N9—C7—N8	125.8 (3)
O8—La1—O7	47.69 (6)	N8—C8—H8A	109.5
O8—La1—O10	67.32 (6)	N8—C8—H8B	109.5
O8—La1—O14	129.20 (7)	N8—C8—H8C	109.5
O8—La1—O16	72.16 (6)	H8A—C8—H8B	109.5
O8—La1—O17	105.15 (6)	H8A—C8—H8C	109.5
O10—La1—O2	67.23 (6)	H8B—C8—H8C	109.5
O10—La1—O5	123.81 (6)	C14—N10—H4A	125.3
O10—La1—O7	113.34 (6)	C15—N10—H4A	125.3
O10—La1—O14	103.01 (6)	C15—N10—C14	109.4 (2)
O10—La1—O16	111.76 (6)	C9—N11—C16	125.3 (3)
O10—La1—O17	158.71 (6)	C15—N11—C9	108.4 (2)
O11—La1—O1	110.99 (6)	C15—N11—C16	126.2 (3)
O11—La1—O2	111.06 (6)	H6A—N12—H6B	120.0
O11—La1—O4	177.33 (6)	C15—N12—H6A	120.0
O11—La1—O5	132.34 (6)	C15—N12—H6B	120.0
O11—La1—O7	108.55 (6)	C10—C9—N11	131.5 (3)
O11—La1—O8	69.00 (6)	C10—C9—C14	122.0 (3)
O11—La1—O10	47.98 (6)	C14—C9—N11	106.6 (3)
O11—La1—O14	69.24 (6)	C9—C10—H10	121.8
O11—La1—O16	67.30 (6)	C9—C10—C11	116.4 (3)
O11—La1—O17	110.92 (6)	C11—C10—H10	121.8
O13—La1—O1	67.53 (7)	C10—C11—H11	119.2
O13—La1—O2	110.77 (7)	C10—C11—C12	121.6 (3)
O13—La1—O4	112.56 (7)	C12—C11—H11	119.2
O13—La1—O5	66.72 (7)	C11—C12—H12	119.0
O13—La1—O7	174.63 (6)	C13—C12—C11	122.1 (3)
O13—La1—O8	132.30 (6)	C13—C12—H12	119.0
O13—La1—O10	68.46 (6)	C12—C13—H13	121.9
O13—La1—O11	68.59 (7)	C12—C13—C14	116.2 (3)
O13—La1—O14	48.26 (6)	C14—C13—H13	121.9
O13—La1—O16	109.55 (7)	N10—C14—C9	106.4 (2)
O13—La1—O17	109.53 (7)	C13—C14—N10	131.9 (3)
O14—La1—O2	158.41 (7)	C13—C14—C9	121.7 (3)
O14—La1—O5	69.15 (6)	N10—C15—N11	109.2 (2)
O14—La1—O7	126.73 (7)	N12—C15—N10	125.5 (3)
O14—La1—O16	65.90 (7)	N12—C15—N11	125.2 (3)
O14—La1—O17	65.41 (6)	N11—C16—H16A	109.5
O16—La1—O2	135.15 (7)	N11—C16—H16B	109.5
O16—La1—O7	65.09 (7)	N11—C16—H16C	109.5
O17—La1—O2	129.64 (7)	H16A—C16—H16B	109.5
O17—La1—O7	66.84 (7)	H16A—C16—H16C	109.5
O17—La1—O16	47.80 (6)	H16B—C16—H16C	109.5
N1—O1—La1	98.69 (16)	C22—N13—H7	125.5
N1—O2—La1	96.49 (15)	C23—N13—H7	125.5
N2—O4—La1	97.46 (15)	C23—N13—C22	109.0 (3)
N2—O5—La1	97.46 (15)	C17—N14—C24	126.6 (3)
N3—O7—La1	95.65 (15)	C23—N14—C17	108.4 (3)
N3—O8—La1	98.41 (14)	C23—N14—C24	125.0 (3)
N4—O10—La1	97.48 (14)	H9A—N15—H9B	120.0
N4—O11—La1	98.06 (16)	C23—N15—H9A	120.0
N5—O13—La1	98.75 (15)	C23—N15—H9B	120.0
N5—O14—La1	96.95 (14)	C18—C17—N14	131.0 (3)
N6—O16—La1	97.36 (15)	C22—C17—N14	106.9 (3)
N6—O17—La1	97.39 (16)	C22—C17—C18	122.1 (3)
O2—N1—O1	116.9 (2)	C17—C18—H18	122.1
O3—N1—O1	120.4 (3)	C17—C18—C19	115.9 (3)
O3—N1—O2	122.7 (3)	C19—C18—H18	122.1
O5—N2—O4	117.1 (2)	C18—C19—H19	119.1
O6—N2—O4	121.3 (2)	C20—C19—C18	121.8 (3)
O6—N2—O5	121.6 (2)	C20—C19—H19	119.1
O8—N3—O7	117.5 (2)	C19—C20—H20	119.2
O9—N3—O7	120.9 (2)	C19—C20—C21	121.7 (3)
O9—N3—O8	121.5 (2)	C21—C20—H20	119.2
O10—N4—O11	115.9 (2)	C20—C21—H21	121.6
O12—N4—O10	122.5 (2)	C22—C21—C20	116.8 (3)
O12—N4—O11	121.7 (3)	C22—C21—H21	121.6
O14—N5—O13	116.0 (2)	C17—C22—N13	106.4 (3)
O15—N5—O13	121.5 (2)	C21—C22—N13	131.9 (3)
O15—N5—O14	122.5 (2)	C21—C22—C17	121.7 (3)
O16—N6—O17	117.3 (2)	N14—C23—N13	109.4 (3)
O18—N6—O16	122.3 (3)	N15—C23—N13	125.4 (3)
O18—N6—O17	120.4 (3)	N15—C23—N14	125.2 (3)
C6—N7—H1	125.5	N14—C24—H24A	109.5
C7—N7—H1	125.5	N14—C24—H24B	109.5
C7—N7—C6	109.0 (2)	N14—C24—H24C	109.5
C1—N8—C8	125.6 (3)	H24A—C24—H24B	109.5
C7—N8—C1	108.7 (2)	H24A—C24—H24C	109.5
C7—N8—C8	125.7 (3)	H24B—C24—H24C	109.5
			
La1—O1—N1—O2	7.3 (3)	N11—C9—C10—C11	178.4 (3)
La1—O1—N1—O3	−171.3 (3)	N11—C9—C14—N10	−0.7 (3)
La1—O2—N1—O1	−7.1 (3)	N11—C9—C14—C13	−179.0 (3)
La1—O2—N1—O3	171.5 (3)	C9—N11—C15—N10	−2.1 (3)
La1—O4—N2—O5	2.2 (2)	C9—N11—C15—N12	179.1 (3)
La1—O4—N2—O6	−175.9 (2)	C9—C10—C11—C12	0.7 (5)
La1—O5—N2—O4	−2.2 (2)	C10—C9—C14—N10	178.5 (3)
La1—O5—N2—O6	175.9 (2)	C10—C9—C14—C13	0.2 (5)
La1—O7—N3—O8	−8.4 (2)	C10—C11—C12—C13	−0.5 (6)
La1—O7—N3—O9	171.0 (2)	C11—C12—C13—C14	0.1 (5)
La1—O8—N3—O7	8.7 (3)	C12—C13—C14—N10	−177.8 (3)
La1—O8—N3—O9	−170.7 (2)	C12—C13—C14—C9	0.0 (4)
La1—O10—N4—O11	7.7 (2)	C14—N10—C15—N11	1.7 (3)
La1—O10—N4—O12	−172.4 (2)	C14—N10—C15—N12	−179.5 (3)
La1—O11—N4—O10	−7.8 (2)	C14—C9—C10—C11	−0.6 (5)
La1—O11—N4—O12	172.4 (2)	C15—N10—C14—C9	−0.6 (3)
La1—O13—N5—O14	−2.3 (2)	C15—N10—C14—C13	177.5 (3)
La1—O13—N5—O15	177.5 (2)	C15—N11—C9—C10	−177.4 (3)
La1—O14—N5—O13	2.3 (2)	C15—N11—C9—C14	1.7 (3)
La1—O14—N5—O15	−177.6 (2)	C16—N11—C9—C10	7.8 (5)
La1—O16—N6—O17	4.3 (2)	C16—N11—C9—C14	−173.1 (3)
La1—O16—N6—O18	−175.8 (2)	C16—N11—C15—N10	172.6 (3)
La1—O17—N6—O16	−4.3 (2)	C16—N11—C15—N12	−6.2 (5)
La1—O17—N6—O18	175.8 (2)	N14—C17—C18—C19	−178.9 (3)
N8—C1—C2—C3	−177.9 (3)	N14—C17—C22—N13	−0.8 (3)
N8—C1—C6—N7	0.0 (3)	N14—C17—C22—C21	178.2 (3)
N8—C1—C6—C5	177.9 (3)	C17—N14—C23—N13	1.2 (3)
C1—N8—C7—N7	−0.3 (3)	C17—N14—C23—N15	−179.4 (3)
C1—N8—C7—N9	−178.4 (3)	C17—C18—C19—C20	0.0 (5)
C1—C2—C3—C4	−0.5 (6)	C18—C17—C22—N13	179.0 (3)
C2—C1—C6—N7	−179.1 (3)	C18—C17—C22—C21	−2.0 (4)
C2—C1—C6—C5	−1.1 (5)	C18—C19—C20—C21	−0.8 (5)
C2—C3—C4—C5	0.2 (7)	C19—C20—C21—C22	0.3 (5)
C3—C4—C5—C6	−0.3 (6)	C20—C21—C22—N13	179.8 (3)
C4—C5—C6—N7	178.1 (3)	C20—C21—C22—C17	1.0 (5)
C4—C5—C6—C1	0.8 (5)	C22—N13—C23—N14	−1.7 (3)
C6—N7—C7—N8	0.3 (3)	C22—N13—C23—N15	178.9 (3)
C6—N7—C7—N9	178.4 (3)	C22—C17—C18—C19	1.4 (4)
C6—C1—C2—C3	0.9 (5)	C23—N13—C22—C17	1.5 (3)
C7—N7—C6—C1	−0.2 (3)	C23—N13—C22—C21	−177.4 (3)
C7—N7—C6—C5	−177.8 (3)	C23—N14—C17—C18	−180.0 (3)
C7—N8—C1—C2	179.1 (3)	C23—N14—C17—C22	−0.2 (3)
C7—N8—C1—C6	0.1 (3)	C24—N14—C17—C18	0.9 (5)
C8—N8—C1—C2	−1.9 (5)	C24—N14—C17—C22	−179.3 (3)
C8—N8—C1—C6	179.1 (3)	C24—N14—C23—N13	−179.7 (3)
C8—N8—C7—N7	−179.2 (3)	C24—N14—C23—N15	−0.3 (5)
C8—N8—C7—N9	2.7 (5)		

### Hydrogen-bond geometry (Å, º)

**Table:**

*D*—H···*A*	*D*—H	H···*A*	*D*···*A*	*D*—H···*A*
C21—H21···O17	0.93	2.64	3.530 (5)	161
C24^i^—H24*C*^i^···O10	0.96	2.52	3.348 (5)	138
N7—H7···O1	0.86	2.01	2.819 (3)	157
N10—H4*A*···O4	0.86	2.05	2.889 (3)	164
N12—H6*A*···O6	0.86	2.10	2.944 (3)	165
N13—H7···O7	0.86	2.11	2.920 (4)	156
N15—H9*B*···O9	0.86	2.14	2.946 (3)	155
N15^ii^—H9*A*^ii^···O17	0.86	2.32	3.001 (3)	136

Symmetry codes: (i) *x*−1, −*y*+1/2, *z*−1/2; (ii) *x*, −*y*+1/2, *z*−1/2.
